# Detection of Fish Allergens in Foods Using an In-House Real-Time PCR Targeting the Ribosomal 18S rRNA Gene

**DOI:** 10.3390/foods11223686

**Published:** 2022-11-17

**Authors:** Simona Cau, Cinzia Daga, Carlo Spanu, Barbara Soro, Tiziana Tedde, Sara Salza, Rita Melillo, Gabriella Piras, Sebastiano Virgilio, Bruna Vodret, Alessandro Graziano Mudadu

**Affiliations:** 1 Veterinary Public Health Institute of Sardinia, Complex Structure of Food Hygiene, Via Duca degli Abruzzi 8, 07100 Sassari, Italy; 2 Department of Veterinary Medicine, University of Sassari, Via Vienna 2, 07100 Sassari, Italy

**Keywords:** fish allergy, ribosomal 18S rRNA, absolute detection, relative detection, food labelling

## Abstract

Fish is one of the major food allergens which, in sensitised individuals, can cause life-threatening allergic reactions, even when present in small amounts. To protect consumers’ health, the correct labeling of foods is important. The objective of the present study was to validate an in-house real-time PCR method targeting the ribosomal 18S rRNA gene as universal DNA marker for the detection of fish in foods. The specificity of the primers was assessed on 20 fish species commonly marketed in the Mediterranean basin and other species of molluscs and crustaceans and foods of animal and plant origin. The absolute detection of the method was assessed using DNA extracted from a fish mixture and the SureFood^®^ QUANTARD Allergen 40 reference material. The relative amount was assessed on a fish and béchamel sauce blend. Commercial food samples either labelled with or without fish in the ingredient list, were tested for the presence of fish DNA. The primer showed high specificity against the selected fish species. The limit of detection (LOD) and limit of quantification (LOQ) of the in-house method were 0.5 pg/µL and 5 pg/µL, respectively. The relative quantification in fish and béchamel blend samples detected a concentration as low as 0.000025%, corresponding to 0.25 mg/kg of fish, indicating the suitability of the method in a food matrix. The presence of fish DNA was always detected in commercial samples in which the presence of fish was listed in the ingredient list. The method was able to detect the presence of fish DNA also in samples in which the presence of fish was indicated as traces or was not declared on the label. The proposed method was demonstrated to be a reliable, specific, and sensitive method for the detection of fish allergens in foods. Therefore, the proposed real-time PCR method could be used as a useful instrument in the verification of compliance with allergen labelling regulations.

## 1. Introduction

Food allergies is a term referring to an immune response directed toward foods [1]. The worldwide prevalence of food allergies has increased in the last 20 years in industrialised and developing countries, affecting up to 3–5% of adults and 10% of children [2,3,4]. Hence, food allergies are a significant public health burden and a major concern in the food sector [5,6]. Fish is among the eight major food allergens, which combined account for over 90% of worldwide reported food allergies [3,7]. Because of its nutritional value (i.e., essential amino acids, vitamins, and ω-3 fatty acids), the consumption of fish has grown worldwide, with a global fish consumption per capita above 20 kg/year in 2018 [8]. This increase has been linked with a corresponding growth of cases of fish allergy [9]. The true fish allergy prevalence is not well established since many factors are involved in its occurrence: geographical and cultural fish consumption habits, the distinction with shellfish reactions, type of food processing, diagnostic methods used, mode of exposure, age, etc. [7,10]. It has been estimated that between 0–7% of the general population worldwide suffers from a fish allergy, depending on the method of diagnosis [11,12,13]. An important risk factor in determining fish allergy is exposure to fish, which is higher in countries with greater fish consumption [10,13]. Fish allergy is generally an Ig-E-mediated reaction, and in fish-sensitised individuals, even a small dose of the allergen can elicit an allergic reaction with the sudden onset of clinical signs [14,15]. A reported reference dose of 27.3 mg of fish protein is indicated to cause allergic reactions in 10% of sensitised patients [16]. Allergic reactions include a wide variety of gastrointestinal (nausea, vomiting, abdominal pain), cutaneous (dermatitis or angioedema), and respiratory (rhinitis and asthma) symptoms to a life-threatening anaphylactic shock [17]. Although several fish proteins have been recognised as potential allergens, parvalbumin (PV) proteins are the major fish allergen reported to cause more than 95% of food allergies associated with fish [10,18,19,20,21]. Two isoforms of parvalbumin have been identified: α-parvalbumins, generally considered nonallergenic, and β-parvalbumin, associated with IgE allergic reactions [22,23,24]. The α- and β-parvalbumin are present in different proportions in fish species, with the first being found mainly in cartilaginous fishes (e.g., sharks and rays) while the latter in bony fishes [25,26]. The content of parvalbumin proteins is higher in white muscle and lower in red muscle, explaining why allergies to cartilaginous fish such as tuna, mackerel, and swordfish are rarely reported, despite being commonly consumed [26,27,28,29]. On the basis of the few available studies conducted to determine the minimal eliciting dose, low amounts (milligrams) of fish could trigger an allergic reaction in sensitised patients [11,16,30]. Despite the continuous effort to define threshold “action levels” or “reference doses” for the major food allergens, in most countries, health agencies still have no set threshold for any of the allergenic foods. Because of the difficulty of setting allergens threshold in foods, any amount of allergic food should be considered at risk of triggering an allergic reaction, even traces present as a consequence of cross contamination (e.g., from raw materials, production lines, or equipment) [14]. Unlike some other allergens, fish allergy is a life-long condition which does not resolve with age; therefore, the strategy of choice to protect the susceptible population is the permanent avoidance of ingestion of fish allergens [7,31,32]. International health authorities and regulatory bodies have focused on risk management strategies for improving consumers’ health and promoting consumers’ awareness through the correct labelling of foods [33,34]. Under the EU legislation [35], the presence of the following allergens in foods should be indicated on the label: fish, crustaceans, molluscs, celery, mustard, sesame seed, gluten, tree nuts, peanuts, milk, eggs, soybean, lupine, and sulphites. With the aim of verifying compliance with labelling regulations and avoiding the unwanted presence of allergens, reliable, specific, and sensitive methods for the identification of allergens in foods are needed [36]. The detection of allergens in foods relies mainly on direct methods, such as the enzyme-linked immunosorbent assay (ELISA), or indirect, DNA-based methods, such as real-time quantitative PCR (qPCR) [37,38,39,40,41,42]. ELISA assays allow the direct detection of parvalbumin proteins; however, they show a low specificity due to cross-reactivity and the influence of food processing on protein structure, which can lead to false positive or negative results [30]. Because of their high specificity and sensitivity, real-time PCRs are reliable methods for the detection and quantification of allergens in processed food and complex food matrices [43,44]. On the other hand, DNA-based methods are more suitable in highly processed foods since they are less affected by thermal treatments, pH alterations, and partial hydrolysis of proteins [38,45]. Even though PCR methods have been mainly used for species authentication purposes, they are a useful tool to detect the potential presence of fish allergens in foods. Real-time PCR assays for the detection of fish have been proposed targeting either nuclear (e.g., parvalbumin, rhodopsin, Hoxc13, and 18s rRNA) and mitochondrial genes (12S rRNA and 16S rRNA) [38,41,46,47,48,49]. Real-time PCR commercial kits for the detection of different allergens are available on the market. However, their specificity should be tested on several closely related fish species to account for possible cross-reaction and on different types of food matrices to account for the possible impact of the preparation process of food products. The overall aim of the present study was to develop an in-house real-time PCR method targeting the 18S rRNA for the detection of several fish species commonly consumed in the Mediterranean region. Fish and nonfish DNA was used to validate the proposed method. Specific objectives of the research were to(a) assess the acceptance criteria of the method (specificity, dynamic range, the limit of detection, and limit of quantification), (b) evaluate the assay selectivity using fish béchamel sauce-spiked samples, and (c) test the suitability of the method on commercial food samples.

## 2. Materials and Methods

### 2.1. Sample Collection and DNA Extraction

Specimens of 20 commercially relevant fish species commonly marketed and consumed in Sardinia (Italy) were purchased as whole fresh specimens at local fish markets and supermarkets. Species identification was based on the morphological identification of fish specimens and labelling requirements of fisheries and aquaculture products introduced by Regulation EU n.1379/2013 [50]. Other nonfish species, including crustaceans, molluscs, and animal and plant species, were purchased to assess the specificity of the method (Table 1). All the samples were ground, homogenised, and stored at −20 °C until analysis. DNA extraction was conducted in duplicates using the Sure Food^®^ Prep Advanced kit (CONGEN, R-Biopharm, Darmstadt, Germany). The concentration and purity of DNA extracted were determined by measuring the absorbance at 260 nm and 280 nm. The DNA extracted from all the samples was stored at −20 °C for subsequent analysis.

### 2.2. Primers and Probe

The RT-PCR-assay detection method was developed targeting the ribosomal 18S rRNA gene. Primers were designed according to Daga et al. [47]: forward primer, 18S F (5′-GTACACACGGCCGGTACAGT-3′), reverse primer 18S R (5′-CATGGGTTTTGGGTCTGATAA-3′), and probe 18S P (FAM-5′-CCGTACTTGGATAACTGTGGCAATTC-3′) (Sigma-Aldrich, St. Louis, MO, USA).

### 2.3. Real-Time PCR

Polymerase chain reaction real-time (qPCR) allows the collection of data throughout the PCR process as it occurs by the combination of amplification and detection into a single step. In a qPCR, a positive reaction is detected by the accumulation of a fluorescent signal. The cycle threshold (CT) value is the number of cycles at which the fluorescent signal generated within a reaction crosses the intensity of background fluorescence. The greater the amount of target nucleic acid, the lower the CT value will be [51,52]. Two different real-time PCR assays were conducted, one using the commercial Sure Food^®^ allergen fish kit (CONGEN, R-Biopharm, Germany) and the second using the in-house method. The commercial real-time kit was used following the manufacturer’s instructions. Briefly, the PCR reactions were carried out in a total volume of 25 µL containing 5 µL of DNA template (50 ng/µL). Conditions of the amplification reaction were: 95 °C for 5 min, 45 cycles at 95 °C for 15 s, and 60 °C for 30 s. The in-house real-time assay was carried out in 20 µL of total reaction volume containing 2 µL of DNA template (50 ng/µL), 10 µL of 2X QuantiTec Multiplex PCR Master Mix (Qiagen, Venlo, The Netherlands), 900 nM of each primer, 250 nM of the probe, and DNA and RNA free water. The amplification reaction was carried out with the following conditions: 95 °C for 15 min, 45 cycles at 95 °C for 15 s, and 60 °C for 1 min. All real-time runs were conducted in duplicate on an ABI Prism 7900 HT Sequence Detection System (Applied Biosystem, Foster City, CA, USA). CT value was generated by 7900 software SDS 2.4 (Applied Biosystems).

### 2.4. Acceptance Criteria of Real-Time PCR Assays

The performance of the two real-time methods was assessed according to the requirements set by the European Network of GMO Laboratories (ENGL) (ENGL, 2015) and the MIQE Guidelines [53].

#### 2.4.1. Specificity

The specificity was defined as the ability of the method to amplify only the target species. The specificity of the selected primers/probe of both in-house and commercial kit real-time PCR methods was tested with 20 fish species of commercial interest. To account for potential cross-reacting species, the specificity was evaluated also using the DNA (50 ng/µL) of nontarget species of molluscs and crustaceans, and foods of animal and plant origin and spices (Table 1). To account for the possible impact of food processing on qPCR specificity, the in-house method was also performed on the following fish species: *Macruronus novaezelandiae* (Blue grenadier), *Merluccius merluccius* (European hake), and *Merluccius paradoxus* (Deep-water Cape hake) before (raw samples) and after cooking (boiling for 15 min) the samples prior to DNA extraction and qPCR.

#### 2.4.2. Dynamic Range

A mixture of five fish species was prepared: *Macruronus novaezelandiae* (Blue grenadier), *Dentex angolensis* (Angola dentex), *Diplodus sargus* (White seabream), *Mullus surmuletus* (Striped red mullet), and *Spicara smaris* (Picarel). Specimens from each fish species were pooled in equal volume, ground, and homogenised to obtain the final mixture. DNA extraction was conducted using the Sure Food^®^ Prep Advanced kit (CONGEN, R-Biopharm, Germany) following instructions. Duplicate pure fish DNA extracts of the mixture, previously submitted to spectrophotometric quantification, were adjusted to a concentration of 50 ng/µL and serially diluted in pure water (from 50,000 pg/µL to 0.125 pg/µL). Each dilution of both extractions was analysed in ten replicates for DNA quantification using the in-house real-time PCR method. To evaluate the amplification efficiency of the qPCR assay, a standard curve was generated by plotting the average CT values (20 replicates) against the log of the corresponding fish DNA quantity in the dilution series. The amplification efficiency (*E*) was calculated with the following equation: E% = [10 (−1/slope) − 1] ×100. The correlation coefficient (R^2^) was calculated from the standard curve obtained by linear regression analysis. The minimum acceptance criteria for real-time PCR detection methods require a slope of the standard curve in the range between −3.1 and −3.6, corresponding to amplification efficiencies of 110% to 90%; the correlation coefficient (R^2^) should be ≥0.98 [53,54].

#### 2.4.3. Limit of Detection (LOD) and Limit of Quantification (LOQ)

The absolute detection of the in-house qPCR targeting the ribosomal 18S rRNA was assessed on the DNA extracted from the fish mixture serially diluted in pure water. Each dilution was analysed in 20 replicates for DNA quantification by in-house real-time PCR. The method performance, limit of detection (LOD), and limit of quantification (LOQ) were determined from the dilution series. The LOD was the last serial dilution amplified in 95% of the replicates; the LOQ was established as the lowest concentration showing a coefficient of variation (CV%) below 25% [54]. The LOD and LOQ were also determined using the reference material SureFood^®^ QUANTARD Allergen 40 (R-biopharm) containing fish and most of the potentially allergenic food ingredients included in regulation (EU) No 1169/2011 in a concentration of 40 mg/kg (ppm) as standard reference fish material. With this aim, the reference material was serially diluted from 40 mg/kg to 0.0125 mg/kg.

#### 2.4.4. Assay Selectivity

The selectivity was defined as the performance of the qPCR assay in the identification and quantification of fish allergens in the presence of interfering substances (i.e., matrix effect). To determine the matrix effect on the performance of the qPCR assay (assay selectivity), the detection of fish targeting the ribosomal 18S rRNA was conducted on a mixture with different concentrations of fish and béchamel sauce (spiked samples). The initial mixture was prepared by adding 50% *w*/*w* of fish mixture in béchamel sauce. The fish mixture (*Macruronus novaezelandiae*, *Dentex angolensis*, *Diplodus sargus*, *Mullus surmuletus,* and *Spicara smaris*) was prepared as previously described in the dynamic range experiment. The béchamel sauce contained whole milk, cream milk, wheat flour, starch, and salt. From the initial 50% fish and béchamel mixture, serial dilutions were prepared using béchamel sauce to obtain the following fish concentrations: 5%, 0.5%, 0.05%, 0.005%, 0.0005%, 0.00005%, 0.000025%, and 0.0000125%. Each dilution was analysed in 10 replicates for DNA quantification with two real-time PCR methods, the in-house qPCR assay and the commercial kit Sure Food^®^ Allergen Fish (CONGEN, R-Biopharm). The performance of the two methods was compared.

#### 2.4.5. Commercial Seafood Samples

After the validation of the in-house method, the applicability of the qPCR was tested on 33 commercially processed seafood obtained from local markets. Samples included 22 types of fish-based foods and eleven seafood-based products, including molluscs, bivalves, and crustaceans. Fish content (%) and, when available, allergen label statements of the presence of fish are also reported. Each sample was analysed in triplicate.

## 3. Results

### 3.1. Specificity

All 20 fish species tested amplified with both qPCR methods, while nontarget DNA samples (nonfish species) were not amplified, indicating no cross-reactivity. Cycle threshold (CT) values (±SD) of the in-house and the commercial real-time PCR test kit for fish allergen, Sure Food^®^ allergen fish kit (CONGEN, R-Biopharm, Germany), are reported in Table 1. Overall, the specificity of the in-house method was comparable with that of the commercial kit. However, for Macruronus novaezelandie and Merluccius merluccius, the higher CT values obtained with the in-house method indicate a lower sensitivity for these two species compared with the commercial kit. The heat treatment (boiling) did not impact the quality of DNA extraction and the specificity of the real-time assay (Supplementary Table S1).

### 3.2. Dynamic Range

**Figure 1:**
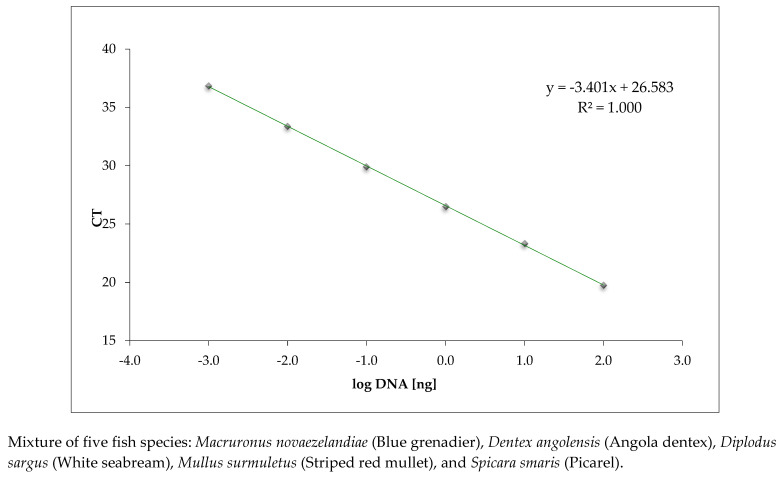
Calibration curve of the in-house real-time PCR method targeting the ribosomal 18S rRNA gene conducted on serially diluted fish DNA extracts.

The dynamic range and linearity are shown in the regression line obtained by plotting the average CT values and the log of the fish DNA quantity in the dilution series (Figure 1). The CT values obtained with the in-house real-time PCR targeting the 18 S rRNA on dilution series of fish DNA extracts are reported in Supplementary Table S2. The calibration curve parameters, namely PCR efficiency (97.0%), R^2^ (1.0), and slope (−3.401), were all within the acceptance criteria, indicating a high performance of the assay.

### 3.3. LOD and LOQ

Table 2 shows the average CT values (±SD), coefficient of variation (CV%), number of positive amplifications on total replicates, and performance parameters obtained from the dilution series of DNA extracted from the fish mixture and the reference material QUANTARD Allergen 40 (SureFood^®^). The average DNA content over which the method performs in a linear manner with an acceptable level of trueness and precision (dynamic range) ranged, respectively, from 50,000 pg/µL to 0.25 pg/µL and from 40 mg/Kg to 0.025 mg/Kg. The LOD, considered the lowest fish DNA concentration, which was amplified in 95% of replicates, was established as 0.5 pg/µL and 0.025 mg/Kg, respectively, for the fish mixture and the reference material. The LOQ was determined as 5 pg/µL and 0.2 mg/Kg for the fish mixture and the reference material, respectively. Performance parameters of the in-house qPCR method, PCR efficiency (E%), correlation coefficient (R^2^), and slope were within the acceptance criteria with both methods.

### 3.4. Assay Selectivity

**Figure 2:**
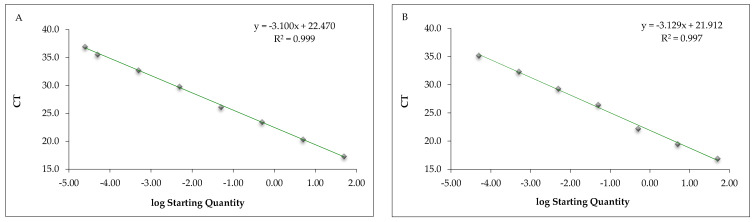
Calibration curve of the real-time PCR in-house method (**A**) and commercial Sure Food^®^ allergen fish kit (**B**) targeting the ribosomal 18S rRNA of fish conducted on serially diluted fish and béchamel sauce mixture.

The mean CT values obtained from the DNA extracted from serial dilution of fish béchamel sauce samples submitted to real-time PCR with the in-house assays and the commercial Sure Food^®^ allergen fish kit (CONGEN) are reported in Table 3, while Figure 2 reports the corresponding calibration curves. The calibration curve parameters were within the acceptance criteria with both methods. The LOD was determined at a fish béchamel sauce concentration of 0.000025%, corresponding to 0.25 mg/kg of fish with the in-house method, and at 0.00005%, corresponding to 0.50 mg/kg of fish with the commercial kit qPCR assays. The LOQ was 0.00005% (0.50 mg/kg) and 0.005% (50 mg/kg), respectively, with the in-house and commercial kit qPCR assays (Table 3).

### 3.5. Applicability of the Method

Overall, 33 food samples were analysed (22 fish-based foods and 10 other types of seafood). The in-house real-time PCR detected the presence of fish in all eighteen fish-based products analysed, indicating high specificity for a wide range of foods. In four out of five seafood samples in which the label declared “fish traces”, the presence of fish allergen was not detected, while one sample (i.e., red clam sauce) was positive. Similarly, in one out of 6 seafood samples (i.e., clams in tomato sauce) in which the presence of fish was not declared, the method detected the presence of fish, suggesting that the method is able to detect the presence of fish traces. The mean CT values ranged between 16.49 ± 0.17 and 34.59 ± 0.17, confirming that the efficiency of the method varies from species to species. Overall CT values and (±SD) are presented in Table 4.

## 4. Discussion

Few studies have been conducted to determine the presence of fish in food samples using real-time PCR methods [38,46,48,55]. The present study developed an in-house real-time PCR method targeting the ribosomal 18S rRNA gene as a universal marker for the detection and quantification of fish in food samples. The amplification was obtained with all selected fish species, indicating that the selected set of primers is suitable for a wide range of fish species. No cross-reaction was observed using nontarget species of molluscs, crustaceans, and foods of animal and plant origin and spices, indicating a high specificity of the method. The verification of the method specificity against the tested ingredients is of relevance since these are frequently used mixed with fish in the preparation of several processed foods. The CT values obtained with the in-house method were comparable with those obtained with the real-time PCR commercial kit Sure Food^®^ Allergen Fish (CONGEN, R-Biopharm), indicating a good specificity of the in-house method. The quality and integrity of DNA extraction were also demonstrated in samples previously submitted to heat treatment, suggesting the potential use of the method in processed foods. The performance of the in-house real-time PCR method was evaluated using serial dilutions of DNA extracted from a fish mixture. The method was performed in the range from 50,000 pg/µL to 0.125 pg/µL of fish DNA, which covered 8 orders of magnitude of the target DNA. The calibration curve generated by plotting the average CT values against the log fish DNA of the dilution series allowed us to evaluate the amplification efficiency (E = 97.0%), the correlation coefficient (R2 = 1.0), and slope (−3.401) of the qPCR assay, which were all within the acceptance criteria for real-time PCR detection methods, demonstrating the applicability of the in-house qPCR method for the detection of fish in foods. The absolute detection (LOD and LOQ) of the in-house real-time PCR was assessed on a serial dilution of DNA extracted from the fish mixture and the SureFood^®^ QUANTARD Allergen 40 reference material. The LOD was established as 0.5 pg/µL and 0.025 mg/Kg for the fish mixture and the reference material, respectively. The LOQ was determined as 5 pg/µL and 0.2 mg/Kg for the fish mixture and the reference material, respectively. Since the presence of other ingredients could affect the performance of the real-time PCR, the method was also tested with samples obtained with fish mixed with different proportions of béchamel sauce. With this aim, the matrix effect was evaluated by comparing the in-house method with the qPCR commercial Sure Food^®^ allergen fish kit (CONGEN). The in-house method showed a greater sensitivity, allowing the quantification of fish DNA at 3 orders of magnitude lower compared with the commercial kit. The LOQ was 0.50 mg/kg (0.00005%) and 50 mg/kg (0.005%), respectively, for the in-house and commercial methods. In addition, the LOD was lower with the in-house method (0.25 mg/kg corresponding to 0.000025%) than with the commercial kit (0.50 mg/kg corresponding to 0.00005%). Of the previous studies which applied real-time PCR for the detection of fish in food samples, only Fernandes et al., 2018 [48] proposed a method suitable to effectively detect and quantify fish at trace levels. Herrero et al., 2014 [38] did not conduct the quantification, while Benedetto et al., 2011 [46] and Pegels et al., 2013 [55] obtained no accurate quantification of fish DNA.

The in-house method developed in the present study showed a considerably lower relative detection of fish DNA in fish and béchamel sauce samples: 0.25 mg/kg compared with Fernandes et al., who obtained a relative detection of 1 mg/kg [9]. The applicability of the qPCR method was tested on 28 commercial processed food products. It can be noticed that when fish was declared on the label as an ingredient, the samples were consistently positive for the presence of the ribosomal 18S rRNA gene, indicating that the selected set of primers is suitable for the identification of the fish species most commonly used in the preparation of foods. The presence of fish was also detected in one sample of clam in tomato sauce where no label indication was provided and in one sample of clam sauce with the indication of fish traces in the label. These findings support the need to accurately verify the presence of unwanted contamination to comply with the allergen label statement. Food allergies are a global food-safety issue; hence the availability of a reliable method for the identification of trace allergens in foods is a critical part of the allergen management program in the food industry. The correct identification of an allergen is necessary to avoid the presence of undeclared and/or unintended allergens in food and meet requirements for allergen labelling. Correct labelling is also important from the consumers’ perspective since the overuse of precautionary labelling (e.g., “may contain…”) could lead to unnecessary dietary restrictions and consumer choice limitations.

## 5. Conclusions

Fish allergies are a major issue for the food industry, and the correct labelling of foods is an integral part of allergen-management strategies. Therefore, the availability of specific and sensitive methods for detecting allergens at trace levels is essential. DNA-based methods have been proposed for the detection of allergens in foods. The present study developed a real-time PCR method for the identification and quantification of fish in food samples based on the ribosomal 18S rRNA gene as a universal marker. The method showed high specificity and sensitivity, demonstrating to be suitable to verify compliance with the labelling requirement of fish allergens in foods.

## Figures and Tables

**Table 1:** Fish and nonfish samples analysed with an in-house and commercial kit * real-time PCR.

Type of Sample	Scientific Name	Common Name	CT ± SD	
			In-House Method	Commercial Kit
Fish	*Macruronus novaezelandie*	Blue grenadier	28.64 ± 0.19	18.86 ± 0.03
	*Merluccius gayi*	Southern Pacific hake	31.41 ± 0.31	29.54 ± 0.14
	*Merluccius merluccius*	European hake	32.70 ± 0.25	21.61 ± 0.13
	*Merluccius paradoxus*	Deep-water Cape hake	31.99 ± 0.60	25.06 ± 0.01
	*Gadus chalcogrammus*	Walleye pollock	34.10 ± 0.95	31.34 ± 0.17
	*Gadus morhua*	Atlantic cod	29.08 ± 0.45	27.49 ± 0.07
	*Dentex Angolensis*	Angola dentex	17.58 ± 0.11	17.21 ± 0.87
	*Diplodus sargus sargus*	White seabream	19.00 ± 0.31	18.65 ± 0.15
	*Sparus aurata*	Gilthead seabream	21.30 ± 0.27	22.78 ± 0.15
	*Scomber scombrus*	Atlantic mackerel	14.88 ± 0.09	16.02 ± 0.03
	*Thunnus alalunga*	Albacore	30.03 ± 0.37	31.59 ± 0.12
	*Salmo salar*	Atlantic salmon	13.38 ± 0.05	14.66 ± 0.08
	*Oncorhynchus gorbuscha*	pink salmon/humpback salmon	15.59 ± 0.23	16.35 ± 0.32
	*Umbrina cirrosa*	Shi drum	21.98 ± 0.72	23.63 ± 0.19
	*Solea solea*	Common sole	17.68 ± 0.21	18.84 ± 0.21
	*Engraulis encrasicolus*	European anchovy	33.68 ± 0.16	33.71 ± 0.15
	*Sardina pilchardus*	Sardine	25.64 ± 0.30	26.83 ± 0.26
	*Mullus surmuletus*	Striped red mullet	18.79 ± 0.03	20.88 ± 0.97
	*Spicara smaris*	Picarel	20.32 ± 0.26	19.41 ± 0.04
	*Trigla lucerna*	tub gurnard	17.58 ± 0.15	18.20 ±0.04
Crustacean	*Penaeus kerathurus*	Caramote prawn	N.D.	N.D.
	*Penaeus monodon*	Giant tiger prawn	N.D.	N.D.
	*Penaeus vannamei*	Whiteleg shrimp	N.D.	N.D.
	*Pandalus borealis*	Northern prawn	N.D.	N.D.
	*Pleoticus muelleri*	Argentine red shrimp	N.D.	N.D.
	*Homarus americanus*	American lobster	N.D.	N.D.
Molluscs	*Todarodes sagittatus*	European flying squid	N.D.	N.D.
	*Loligo forbesi*	Veined squid	N.D.	N.D.
	*Octopus vulgaris*	Common octopus	N.D.	N.D.
	*Mytilus galloprovincialis*	Mediterranean mussel	N.D.	N.D.
	*Crassostrea gigas*	Pacific cupped oyster	N.D.	N.D.
	*Ruditapes decussatus*	Grooved carpet shell (clam)	N.D.	N.D.
Animal- and plant-origin foods	*Bos taurus*	Beef	N.D.	N.D.
	*Sus scrofa domesticus*	Pork	N.D.	N.D.
	*Gallus gallus*	Chicken egg	N.D.	N.D.
	*Apium graveolens var. dulce*	Celery	N.D.	N.D.
	*Petroselinum sativum*	Parsley	N.D.	N.D.
	*Triticum aestivum*	Wheat	N.D.	N.D.
	*Origanum vulgare*	Oregano	N.D.	N.D.
	*Salvia officinalis*	Sage	N.D.	N.D.
	*Thymus vulgaris*	Thyme	N.D.	N.D.
	*Laurus nobilis*	Bay	N.D.	N.D.
	*Piper nigrum*	Black Pepper	N.D.	N.D.

* Sure Food^®^ allergen fish kit (CONGEN, R-Biopharm, Germany); CT—Cycle threshold; N.D.—Not Detected. CT values are the mean of triplicate samples.

**Table 2:** Limit of detection (LOD) and limit of quantification (LOQ) of an in-house real-time PCR for the detection of fish targeting the ribosomal 18S rRNA gene conducted on DNA extracted from a fish mixture and the SureFood^®^ QUANTARD Allergen 40 reference material.

DNA Concentration	Limit of Quantification (LOQ) ^a^		Limit of Detection (LOD) ^d^		
	CT ± SD ^b^	CV % ^c^	Fish Positive	Fish Negative	Detection Rate (%)
Fish mix (pg/µL)					
50,000	19.75 ± 0.34	24	20/20	0/20	100
5000	23.33 ± 0.34	22	20/20	0/20	100
500	26.49 ± 0.33	23	20/20	0/20	100
50	29.91 ± 0.29	20	20/20	0/20	100
5	33.38 ± 0.38	25	20/20	0/20	100
0.5	36.84 ± 0.89	43	20/20	0/20	100
0.25	39.84 ± 2.18	92	15/20	5/20	75
0.125	40.31 ± 0.92	58	4/20	16/20	20
PCR efficiency (%)	97.0%				
Correlation coefficient (R^2^)	1.00				
Slope	−3.401				
Reference material (mg/kg)					
40	24.44 ± 0.17	11	20/20	0/20	100
4	28.17 ± 0.45	23	20/20	0/20	100
0.4	31.50 ± 0.28	19	20/20	0/20	100
0.2	32.48 ± 0.31	18	20/20	0/20	100
0.1	33.37 ± 0.45	26	20/20	0/20	100
0.05	35.20 ± 0.41	26	20/20	0/20	100
0.025	37.46 ± 1.34	70	19/20	1/20	95
0.0125	40.21 ± 0.86	49	4/20	16/20	20
PCR efficiency (%)	91.1%				
Correlation coefficient (R^2^)	0.995				
Slope	−3.556				

^a^ Limit of quantification (LOQ)—lowest DNA concentration showing CV ≤ 25%; ^b^ CT ± SD—cycle threshold ± standard deviation (SD); ^c^ CV—coefficient of variation; ^d^ Limit of Detection (LOD)—last DNA dilution detected in 95% of replicates. Within brackets, positive amplification/total replicates.

**Table 3:** Limit of detection (LOD) and limit of quantification (LOQ) of fish and béchamel sauce dilution series (spiked samples) performed with both in-house and commercial kit ^a^ real-time PCR for the detection of fish targeting the ribosomal 18S rRNA (10 replicates).

Fish Relative Amount (% *w*/*w* Fish-Béchamel)	Limit of Quantification ^b^ (LOQ)				Limit of Detection ^c^ (LOD)					
	In-House qPCR		Commercial Kit		In-House qPCR			Commercial Kit		
	CT ± SD	CV % ^d^	CT ± SD	CV %	Fish Positive	Fish Negative	Detection Rate (%)	Fish Positive	Fish Negative	Detection Rate (%)
50	17.29 ± 0.15	11	16.93 ± 0.26	22	10/10	0/10	100	10/10	0/10	100
5	20.34 ± 0.14	10	19.47 ± 0.24	16	10/10	0/10	100	10/10	0/10	100
0.5	23.43 ± 0.12	9	22.20 ± 0.35	22	10/10	0/10	100	10/10	0/10	100
0.05	26.11 ± 0.19	14	26.46 ± 0.20	14	10/10	0/10	100	10/10	0/10	100
0.005	29.82 ± 0.34	24	29.32 ± 0.25	20	10/10	0/10	100	10/10	0/10	100
0.0005	32.72 ± 0.29	24	32.33 ± 0.39	28	10/10	0/10	100	10/10	0/10	100
0.00005	35.62 ± 0.35	25	35.18 ± 0.63	38	10/10	0/10	100	10/10	0/10	100
0.000025	36.94 ± 0.35	29	41.32 ± 1.17	56	10/10	0/10	100	4/10	6/10	40
0.0000125	40.59 ± 0.79	68	N.D.	N.D.	4/10	6/10	40	N.D.	N.D.	N.D.

^a^ Sure Food^®^ Allergen Fish (CONGEN. R-Biopharm); ^b^ Limit of Quantification (LOQ)—lowest DNA concentration showing CV ≤ 25%. ^c^ Limit of Detection (LOD)—last DNA dilution detected in 95% of replicates; ^d^ CV—coefficient of variation. N.D.—Not Detected.

**Table 4:** Identification of fish allergens by real-time PCR targeting the ribosomal 18S rRNA in commercial seafood.

Type of Food		Allergen Label Statement *	CT ± SD
Fish-based foods	Sliced fish meat sausage	Fish flesh 38%	21.29 ± 0.02
	Breaded anchovies	Anchovies 56%	29.76 ± 0.03
	Frozen breaded fish sticks	Cod fillets 65%	27.32 ± 0.18
	Crab-flavoured surimi stick	Fish flesh 30%	16.49 ± 0.17
	Fish granulated bouillon	Dried cod powder 14%	29.94 ± 0.52
	Fish burger	Alaskan cod 52%	29.72 ± 0.23
	Cod fillets	Alaskan cod 52%	25.41 ± 0.23
	Fish mousse	Albacore 36%	27.53 ± 0.04
	Sardines in tomato sauce	Sardines 73%	25.63 ± 0.01
	Fish soup	Fish mix	26.05 ± 0.48
	Smoked salmon fish sticks	Fish flesh 43% (Salmon 8%)	24.62 ± 0.24
	Cod burger	Cod 85%	29.49 ± 0.12
	Tuna sauce	Tuna 19.5%	33.68 ± 0.16
	Surimi breaded crab claws	Fish flesh 45%	22.30 ± 0.13
	Cod sticks	Cod 65%	29.33 ± 0.07
	Salmon Patè	Salmon 38%	33.65 ± 0.19
	Fish nuggets	Cod 30%	30.56 ± 0.18
	Surimi sticks	Fish flesh 38%	18.95 ± 0.22
	Fish broth cubes	Cod 4.9%	29.68 ± 0.20
	Fish-flavoured bouillon mix	Fish mix 35%	26.80 ± 0.06
	Fish burger	Fish 52%	17.80 ± 0.16
	Salmon sticks	Salmon 65%	16.35 ± 0.09
Other Seafood	Frozen squid rings	trace	N.D.
	Seafood salad	trace	N.D.
	Red clam sauce	trace	33.31 ± 0.20
	Squid in tomato sauce	trace	N.D.
	Black rice and shrimps ready meal	trace	N.D.
	Tail-off shrimp	not declared	N.D.
	Clams in tomato sauce	not declared	34.59 ± 0.17
	Seafood tomato sauce	not declared	N.D.
	Seafood salad in oil	not declared	N.D.
	Breaded squid rings	not declared	N.D.
	Seafood mix	not declared	N.D.

* Allergen label statement refers to the declaration of the presence of fish. CT ± SD—cycle threshold ± standard deviation (SD); N.D.—not detected.

## Data Availability

Data is contained within the article or supplementary material.

## Supplementary Materials

The following supporting information can be downloaded at: https://www.mdpi.com/article/10.3390/foods11223686/s1, Table S1: comparison of mean CT values obtained with the in-house real-time PCR on DNA extracted from raw and cooked fish samples; Table S2: in-house real-time PCR targeting the 18S rRNA on dilution series of DNA extracted from the fish mixture.
