# Supplementary material for: Detection of Fish Allergens in Foods Using an In-House Real-Time PCR Targeting the Ribosomal 18S rRNA Gene
Source: Foods. 2022 Nov 17;11(22):3686. doi: 10.3390/foods11223686 (PMC9689354; doi:10.3390/foods11223686)
Supplement: Supplementary file 1 [file foods-11-03686-s001.zip › foods-2011860-supplementary.pdf]

Supplementary Table S1. Comparison of mean CT values obtained with the in-house real-time PCR on DNA extracted from raw and cooked fish samples.

Fish sample		CT±SD	
Scientific name	Common name	raw samples	cooked samples*
Macruronus novaezelandie	Blue grenadier	28.64 ± 0.19	28.09 ± 0.03
Merluccius merluccius	European hake	32.70 ± 0.25	31.81 ± 0.03
Merluccius paradoxus	Deep-water Cape hake	31.99 ± 0.60	29.92 ± 0.07

CT = Cicle Threshold; *boiling for 15 min. CT values are the mean of 3 replicates.

Supplementary Table S2. In-house real-time PCR targeting the 18S rRNA on dilution series of DNA extracted from fish mixture*.

Fish DNA concentration (ng/µL)															
50		5		0.5		0.05		0.005		0.0005		0.00025		0.000125	
CT (A)	CT (B)	CT (A)	CT (B)	CT (A)	CT (B)	CT (A)	CT (B)	CT (A)	CT (B)	CT (A)	CT (B)	CT (A)	CT (B)	CT (A)	CT (B)
18.94	19.81	22.90	23.38	26.25	26.70	29.82	30.31	33.37	33.36	36.36	38.80	43.80	38.52	41.42	N.D.
19.37	19.78	23.20	23.05	26.18	26.57	30.36	29.90	32.94	33.78	37.38	37.37	39.00	N.D.	N.D.	36.69
19.28	19.76	22.91	23.22	26.16	26.59	29.57	30.08	32.63	33.32	35.88	37.68	41.89	37.96	N.D.	N.D.
19.53	20.29	23.27	23.33	25.83	26.79	29.66	29.66	33.06	32.80	36.17	36.99	40.86	38.58	N.D.	N.D.
19.74	20.01	22.86	23.45	26.98	26.71	29.71	30.06	33.41	33.54	36.23	36.09	N.D.	N.D.	N.D.	N.D.
20.13	20.15	23.57	23.90	27.15	26.73	29.95	30.31	33.32	33.55	37.89	37.88	39.49	40.84	N.D.	40.65
19.88	19.81	23.57	24.02	26.06	26.71	29.75	30.17	34.36	33.81	36.92	36.27	41.76	37.85	N.D.	N.D.
19.69	19.74	23.45	23.87	26.48	26.63	29.97	30.15	33.36	33.72	36.87	36.12	N.D.	37.60	N.D.	N.D.
19.66	20.22	23.01	23.41	26.09	26.55	29.28	29.92	33.08	33.21	36.02	36.69	42.37	N.D.	N.D.	N.D.
19.39	19.89	23.10	23.03	26.20	26.50	29.47	30.05	33.39	33.51	36.62	36.66	39.57	37.44	N.D.	39.48

* *Macruronus novaezelandiae*, *Dentex angolensis*, *Diplodus sargus, Mullus surmuletus* and *Spicara* smaris. Columns (A) and (B) are the dilution series of the duplicate DNA extraction from fish-mixture; each row represents one of the 10 technical replicates. N.D. = not detected. CT = Cicle Threshold;
